# Epalrestat tetra­hydro­furan monosolvate: crystal structure and phase transition

**DOI:** 10.1107/S2056989017007976

**Published:** 2017-06-02

**Authors:** Daiki Umeda, Okky Dwichandra Putra, Mihoko Gunji, Kaori Fukuzawa, Etsuo Yonemochi

**Affiliations:** a School of Pharmacy and Pharmaceutical Sciences, Hoshi University, 2-4-41, Ebara, Shinagawa, Tokyo 145-8501, Japan

**Keywords:** crystal structure, epalerstat, tetra­hydro­furan, monosolvate, hydrogen bonding

## Abstract

The title compound, epalrestat {systematic name: (5*Z*)-5-[(2*E*)-2-methyl-3-phenyl­prop-2-en-1-yl­idene]-4-oxo-2-sulfanyl­idene-1,3-thia­zolidine-3-acetic acid}, crystallized as a tetra­hydro­furan monosolvate, C_15_H_13_NO_3_S_2_·C_4_H_8_O. Epalrestat, an important drug for diabetic neuropathy, has been reported to exist in polymphic, solvated and co-crystal forms. In the mol­ecule reported here, the phenyl ring is inclined to the rhodamine ring by 22.31 (9)°, and the acetic acid group is almost normal to the rhodamine ring, making a dihedral angle of 88.66 (11)°. In the crystal, pairs of O—H⋯O hydrogen bonds are observed between the carb­oxy­lic acid groups of epalerstat mol­ecules, forming inversion dimers with an *R*_2_^2^(8) loop. The dimers are linked by pairs of C—H⋯O hydrogen bonds, forming chains along [101]. The solvate mol­ecules are linked to the chain by a C—H⋯O(tetra­hydro­furan) hydrogen bond. A combination of thermal analysis and powder X-ray diffraction revealed that title compound desolvated into epalerstat Form II. One C atom of the tetra­hydro­furan solvate mol­ecule is positionally disordered and has a refined occupancy ratio of 0.527 (18):0.473 (18).

## Abstract 2

Epalrestat, an important drug for diabetic neuropathy, has been reported to exist in polymphic, solvated and co-crystal forms. Herein, we report on the crystal structure of epalerstat tetra­hydro­furan solvate which crystallizes in the triclinic space group *P* 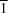. On desolvation, epalerstat Form II (monoclinic, *C*2/*c*) is obtained.

## Chemical context   

Solid-state characterization is an important aspect in the regulation and development as well as intellectual property matter of drugs. Its necessity is based on the requirement to determine the solid-state structure of the drugs because pharmaceutical materials have the ability to exist in various forms, such as polymorphs, salts, co-crystals, and solvates (Putra *et al.*, 2016*a* ▸,*b* ▸). An important class of pharmaceutical materials is solvates, which are defined as being a crystalline multi-component system in which a solvent(s) is accommodated within the crystal structure in a stochiometric or non-stochiometric manner (Griesser, 2006 ▸). Over the past decades, many different solvates with readily discernible physicochemical properties and marked differences in their performances have been reported (Iwata *et al.*, 2014 ▸; Furuta *et al.*, 2015 ▸). Different solvate formations play a significant role in drug development because of their physical instability and the potential toxicity from the solvent mol­ecules. In addition, a tendency to form a solvate sometimes limits the number of solvents available for drug development and manufacturing processes (Campeta *et al.*, 2010 ▸). Therefore, the study of solvate formation is extremely important for the pharmaceutical industry.
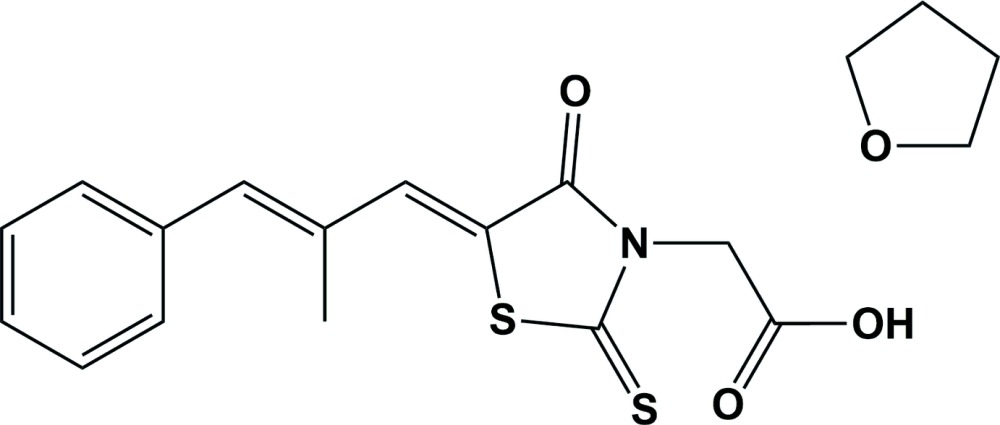


Epalerstat is an aldose reductase inhibitor and is used for the treatment of diabetic neuropathy, which is one of the most common long-term complications in patients with diabetes mellitus. The mechanism of epalerstat is thought to inhibit the first enzyme in the polyol pathway, which converts glucose to sorbitol. Sorbitol itself has been considered to be the cause for diabetic complications including diabetic neuropathy (Miyamoto, 2002 ▸; Ramirez & Borja, 2008 ▸). The solid-state forms of epalerstat as well as their properties have been widely investigated.

It is known that this drug exists in five polymorphic forms, of which three polymorphic structures have been determined by single crystal X-ray structure analysis and two forms have been characterized by spectroscopic methods. The three crystal forms are: Form I (triclinic, *P* 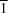; Igarashi *et al.*, 2013 ▸; Swapna *et al.*, 2016 ▸), Form II (monoclinic, *C*2/*c*), and Form III (monoclinic, *P*2_1_/*c*; Swapna *et al.*, 2016 ▸). In addition, the *Z*,*Z* isomer of epalerstat has been determined crystallographically (Swapna *et al.*, 2016 ▸). It has also been reported to exist in multi-component crystal forms, such as solvates with ethanol (Ishida *et al.*, 1990 ▸), methanol (Igarashi *et al.*, 2015 ▸), methanol disolvate (Nagase *et al.*, 2016 ▸), di­methyl­formamide, di­methyl­sulfoxide and as a co-crystal with caffeine (Putra *et al.*, 2017 ▸). The occurrence of solvated epalerstat crystals themselves is not unexpected owing to the imbalance between the potential donors and acceptors of hydrogen bonds in the epalerstat structure. In the present study, we report on the crystal structure of epalerstat in a new solvated form (tetra­hydro­furan monosolvate), and on its thermal behaviour by different physicochemical methods.

## Structural commentary   

**Figure 1:**
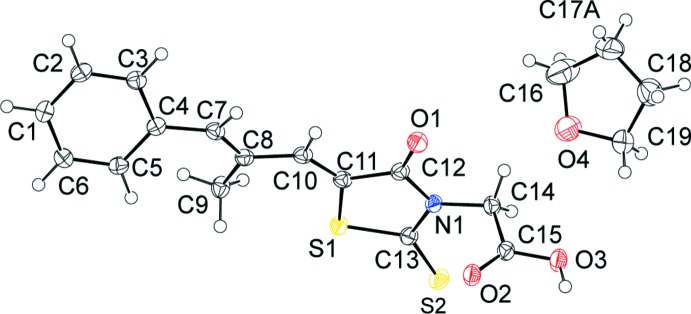
The mol­ecular structure of the title compound, with the atom labelling and displacement ellipsoids drawn at the 50% probability level. The minor disorder component of the solvent molecule is not shown for clarity.

The mol­ecular structure of epalerstat tetra­hydro­furan monosolvate is illustrated in Fig. 1 ▸. The values of all bond distances and angles, and dihedral angles appear to be within normal limits according to the *Mogul* geometry check within the CSD software (Bruno *et al.*, 2004 ▸; CSD, Version 5.38, update February 2017; Groom *et al.*, 2016 ▸). The phenyl ring is inclined to the five-membered ring of the rhodamine unit (N1/S1/C11–C13) by 22.31 (9)°. The acetic acid group (C14/C15/O2/O3) is almost normal to five-membered ring of the rhodamine unit with a dihedral angle of 88.66 (11)°. In addition, the mean plane of the methyl­propenyl­idene (C7–C10) unit is inclined to the phenyl and rhodamine rings by 29.43 (11) and 9.19 (11)°, respectively.

## Supra­molecular features   

**Figure 2:**
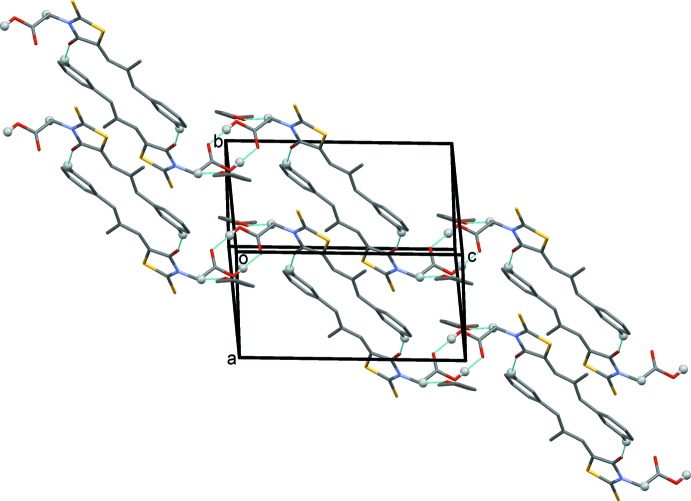
A view normal to (110) of the crystal structure of epalerstat tetra­hydro­furan monosolvate. Hydrogen bonds are shown as dashed lines (see Table 1 ▸) and only H atoms involved in these inter­actions have been included.

In the crystal, each epalerstat mol­ecule is connected to two other epalerstat mol­ecules and one tetra­hydro­furan mol­ecule by both conventional and non-conventional hydrogen bonds. Numerical details of the hydrogen bonds are listed in Table 1 ▸ and are illustrated in Fig. 2 ▸. A pair of O—H⋯O hydrogen bonds is observed between the carb­oxy­lic moieties of epalerstat mol­ecules, forming an inversion dimer with an 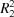
(8) loop. The dimers are linked by pairs of C—H⋯O hydrogen bonds, forming chains along [101]. The solvate mol­ecules are linked to the chain by a C—H⋯O_t_ (t = THF) hydrogen bond.

## Phase transition – thermal behaviour and powder X-ray diffraction   

**Figure 3:**
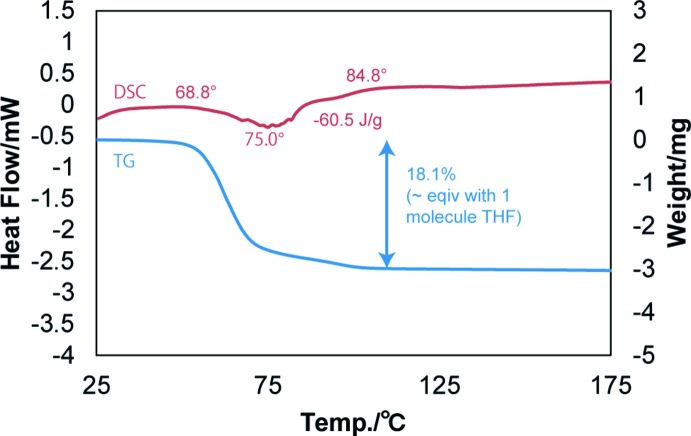
The TG–DSC scan of epalerstat tetra­hydro­furan monosolvate.

In order to understand the thermal behaviour of this solvate at elevated temperatures, the sample was investigated by thermal gravimetry–differential scanning calorimetry (TG–DSC) and powder X-ray diffraction–differential scanning calorimetry (PXRD–DSC) methods (Figs. 3 ▸ and 4 ▸). The TG–DSC measurement was performed in the temperature region from room temperature to 448 K at a rate of 3 K min^−1^. In addition, the PXRD–DSC measurement was conducted from room temperature to 383 K at a heating rate of 3 K min^−1^.

The mass loss started from 341.8–357.5 K and the onset peak appeared at 348 K. The total mass loss was observed to be 18.1%, which is almost equivalent to the loss of one mol­ecule of tetra­hydro­furan (the theoretical value corresponding to one tetra­hydro­furan mol­ecule is 18.4%). Therefore, the occupancy of the solvent mol­ecule was fixed at 1 during crystal-structure refinement. The mass loss corresponds to the desolvation process indicated by the existence of a broad endothermic peak, which occurs in the DSC thermogram at a similar temperature. The enthalpy of desolvation was estimated to be −60.5 J g^−1^(8.3 × 10^−4^ kJ mol^−1^).

In order to understand the phase transformation during the heating, a PXRD–DSC measurement was carried out. The desolvation temperature observed by PXRD–DSC was slightly different compared to the TG-DSC measurement. The desolvation started from 303–343 K in this case. The differences in temperature derived from TG–DSC and PXRD–DSC seem to be reasonable due the differences in the experimental conditions of both the methods. A closed pan system was used in the TG–DSC measurement, while an open pan system was applied in the PXRD–DSC measurement. By comparing the powder X-ray diffractogram to those for the reported polymorphic forms of epalerstat, it was seen that epalerstat tetra­hydro­furan monosolvate desolvated into epalerstat.

## Database survey   

A search of the Cambridge Structural Database (Version 5.38, update February 2017; Groom *et al.*, 2016 ▸) for epalerstat yielded nine hits. They include, the methanol disolvate (EHEQUF; Nagase *et al.*, 2016 ▸), the *Z*,*Z* isomer (LALZEG; Swapna *et al.*, 2016 ▸), the ethanol solvate (SALVIK; Ishida *et al.*, 1989 ▸; SALVIK10; Ishida *et al.*, 1990 ▸), the methanol monosolvate (XUBVOH; Igarashi *et al.*, 2015 ▸), and Form I: triclinic, *P* 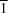 (ZIPKOA; Igarashi *et al.*, 2013 ▸; ZIPKOA3; Swapna *et al.*, 2016 ▸), Form II: monoclinic, *C*2/*c* (ZIPLOA02; Swapna *et al.*, 2016 ▸) and Form III: monoclinic, *P*2_1_/*n* (ZIPKOA01; Swapna *et al.*, 2016 ▸).

## Synthesis and crystallization   

Epalerstat form I (700 mg) was dissolved in tetra­hydro­furan (10 ml) and the solution was kept for one week at room temperature, after which yellow plate-like crystals of the title compound were obtained.

## Refinement details   

Crystal data, data collection and structure refinement details are summarized in Table 2 ▸. The OH H atom was located in a difference-Fourier map and freely refined. The C-bound H atoms were included in calculated positions and treated as riding: C—H = 0.9–1.0 Å with *U*_iso_(H) = 1.5*U*_iso_(C-meth­yl) and 1.2*U*_iso_(C) for other H atoms. One C atom (C17) of the tetra­hydro­furan mol­ecule is positionally disordered and has a refined occupancy ratio (C17*A*:C17*B*) of 0.527 (18):0.473 (18).

## Supplementary Material

Crystal structure: contains datablock(s) I, global. DOI: 10.1107/S2056989017007976/su5372sup1.cif

Structure factors: contains datablock(s) I. DOI: 10.1107/S2056989017007976/su5372Isup2.hkl

Supporting information file. DOI: 10.1107/S2056989017007976/su5372Isup3.cml

CCDC reference: 1553010

Additional supporting information:  crystallographic information; 3D view; checkCIF report

## Figures and Tables

**Figure 4:**
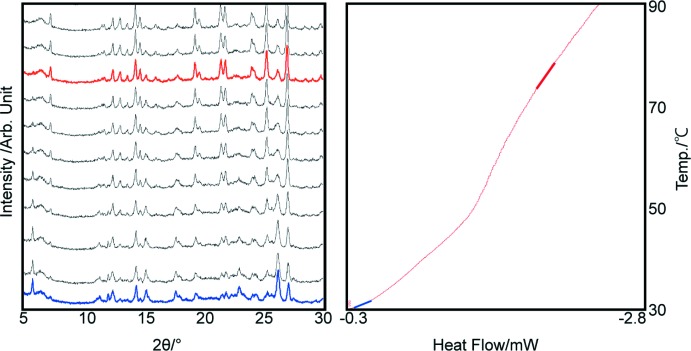
The PXRD–DSC scan of epalerstat tetra­hydro­furan monosolvate. The blue and red PXRD patterns represent the epalerstat tetra­hydro­furan monosolvate and epalerstat form II, respectively.

**Table 1:** Hydrogen-bond geometry (Å, °)

*D*—H⋯*A*	*D*—H	H⋯*A*	*D*⋯*A*	*D*—H⋯*A*
O3—H3*O*⋯O2^i^	0.92 (3)	1.73 (3)	2.6440 (18)	175 (3)
C14—H14*B*⋯O4	0.99	2.26	3.127 (2)	145
C2—H2⋯O1^ii^	0.95	2.51	3.389 (2)	154

Symmetry codes: (i) 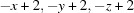; (ii) 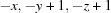.

**Table 2:** Experimental details

Crystal data	
Chemical formula	C_15_H_13_NO_3_S_2_·C_4_H_8_O
*M*_r_	391.49
Crystal system, space group	Triclinic, *P* 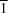
Temperature (K)	93
*a*, *b*, *c* (Å)	7.8956 (3), 8.9627 (3), 15.0311 (4)
α, β, γ (°)	102.263 (7), 93.970 (7), 114.219 (8)
*V* (Å^3^)	933.23 (8)
*Z*	2
Radiation type	Cu *K*α
μ (mm^−1^)	2.80
Crystal size (mm)	0.44 × 0.33 × 0.12

Data collection	
Diffractometer	RIGAKU R-AXIS RAPID II
Absorption correction	Multi-scan (*ABSCOR*; Higashi, 1995 ▸)
*T*_min_, *T*_max_	0.365, 0.721
No. of measured, independent and observed [*I* > 2σ(*I*)] reflections	10947, 3342, 3184
*R*_int_	0.029
(sin θ/λ)_max_ (Å^−1^)	0.602

Refinement	
*R*[*F* ^2^ > 2σ(*F* ^2^)], *wR*(*F* ^2^), *S*	0.036, 0.097, 1.03
No. of reflections	3342
No. of parameters	250
H-atom treatment	H atoms treated by a mixture of independent and constrained refinement
Δρ_max_, Δρ_min_ (e Å^−3^)	0.54, −0.41

Computer programs: *PROCESS-AUTO* (Rigaku, 1998 ▸), *SHELXS2014* (Sheldrick, 2008 ▸), *Mercury* (Macrae *et al.*, 2008 ▸), *SHELXL2016* (Sheldrick, 2015 ▸), *PLATON* (Spek, 2009 ▸) and *publCIF* (Westrip, 2010 ▸).

## supplementary crystallographic information

### Crystal data

**Table:**

C_15_H_13_NO_3_S_2_·C_4_H_8_O	*Z* = 2
*M**_r_* = 391.49	*F*(000) = 412
Triclinic, *P*1	*D*_x_ = 1.393 Mg m^−^^3^
*a* = 7.8956 (3) Å	Cu *K*α radiation, λ = 1.54187 Å
*b* = 8.9627 (3) Å	Cell parameters from 10947 reflections
*c* = 15.0311 (4) Å	θ = 3.1–68.2°
α = 102.263 (7)°	µ = 2.80 mm^−^^1^
β = 93.970 (7)°	*T* = 93 K
γ = 114.219 (8)°	Plate, yellow
*V* = 933.23 (8) Å^3^	0.44 × 0.33 × 0.12 mm

### Data collection

**Table:**

RIGAKU R-AXIS RAPID II diffractometer	3342 independent reflections
Radiation source: Rotating Anode X-ray, RIGAKU	3184 reflections with *I* > 2σ(*I*)
Detector resolution: 10.0 pixels mm^-1^	*R*_int_ = 0.029
ω scan	θ_max_ = 68.2°, θ_min_ = 3.1°
Absorption correction: multi-scan (ABSCOR; Higashi, 1995)	*h* = −9→9
*T*_min_ = 0.365, *T*_max_ = 0.721	*k* = −10→10
10947 measured reflections	*l* = −18→17

### Refinement

**Table:**

Refinement on *F*^2^	Primary atom site location: structure-invariant direct methods
Least-squares matrix: full	Secondary atom site location: difference Fourier map
*R*[*F*^2^ > 2σ(*F*^2^)] = 0.036	Hydrogen site location: mixed
*wR*(*F*^2^) = 0.097	H atoms treated by a mixture of independent and constrained refinement
*S* = 1.03	*w* = 1/[σ^2^(*F*_o_^2^) + (0.0508*P*)^2^ + 0.6912*P*] where *P* = (*F*_o_^2^ + 2*F*_c_^2^)/3
3342 reflections	(Δ/σ)_max_ = 0.001
250 parameters	Δρ_max_ = 0.54 e Å^−^^3^
0 restraints	Δρ_min_ = −0.41 e Å^−^^3^

### Special details

**Table:**

Geometry. All esds (except the esd in the dihedral angle between two l.s. planes) are estimated using the full covariance matrix. The cell esds are taken into account individually in the estimation of esds in distances, angles and torsion angles; correlations between esds in cell parameters are only used when they are defined by crystal symmetry. An approximate (isotropic) treatment of cell esds is used for estimating esds involving l.s. planes.

### Fractional atomic coordinates and isotropic or equivalent isotropic displacement parameters (Å^2^)

**Table:**

	*x*	*y*	*z*	*U*_iso_*/*U*_eq_	Occ. (<1)
S1	0.90767 (6)	0.79772 (6)	0.58395 (3)	0.02165 (13)	
S2	1.23488 (6)	0.84882 (6)	0.71815 (3)	0.02648 (14)	
O1	0.54949 (17)	0.57699 (17)	0.72847 (9)	0.0272 (3)	
O2	0.91598 (19)	0.92945 (16)	0.88861 (8)	0.0262 (3)	
O3	1.0248 (2)	0.80276 (18)	0.97606 (9)	0.0284 (3)	
H3O	1.040 (4)	0.892 (4)	1.024 (2)	0.057 (8)*	
O4	0.6533 (3)	0.4293 (2)	0.93062 (11)	0.0564 (5)	
N1	0.8689 (2)	0.69156 (18)	0.73069 (9)	0.0196 (3)	
C1	0.0117 (3)	0.7933 (2)	0.23383 (12)	0.0256 (4)	
H1	−0.051735	0.810846	0.183855	0.031*	
C2	−0.0869 (3)	0.6655 (2)	0.27480 (12)	0.0250 (4)	
H2	−0.217761	0.595502	0.253129	0.030*	
C3	0.0074 (2)	0.6411 (2)	0.34754 (12)	0.0221 (4)	
H3	−0.061146	0.555352	0.376304	0.026*	
C4	0.2013 (2)	0.7396 (2)	0.37977 (12)	0.0202 (4)	
C5	0.2977 (2)	0.8696 (2)	0.33820 (12)	0.0228 (4)	
H5	0.428474	0.940352	0.359631	0.027*	
C6	0.2027 (3)	0.8951 (2)	0.26601 (13)	0.0248 (4)	
H6	0.269248	0.983274	0.238333	0.030*	
C7	0.2896 (2)	0.7040 (2)	0.45644 (12)	0.0213 (4)	
H7	0.207244	0.655152	0.496164	0.026*	
C8	0.4705 (2)	0.7296 (2)	0.47979 (12)	0.0206 (4)	
C9	0.6252 (3)	0.7977 (3)	0.42567 (12)	0.0247 (4)	
H9A	0.569547	0.783399	0.362311	0.037*	
H9B	0.702595	0.735539	0.424348	0.037*	
H9C	0.704146	0.918410	0.455081	0.037*	
C10	0.5121 (2)	0.6831 (2)	0.56248 (12)	0.0205 (4)	
H10	0.405767	0.630379	0.589848	0.025*	
C11	0.6774 (2)	0.7027 (2)	0.60690 (12)	0.0202 (4)	
C12	0.6825 (2)	0.6480 (2)	0.69292 (12)	0.0209 (4)	
C13	1.0069 (2)	0.7763 (2)	0.68538 (11)	0.0205 (4)	
C14	0.9120 (3)	0.6611 (2)	0.81891 (12)	0.0219 (4)	
H14A	1.023556	0.636832	0.819487	0.026*	
H14B	0.804135	0.560647	0.826837	0.026*	
C15	0.9511 (2)	0.8128 (2)	0.89771 (12)	0.0216 (4)	
C16	0.5125 (4)	0.2659 (4)	0.88746 (18)	0.0598 (8)	
H23A	0.567974	0.194312	0.854014	0.072*	0.527 (18)
H23B	0.417894	0.270682	0.842774	0.072*	0.527 (18)
H23C	0.549421	0.216468	0.831071	0.072*	0.473 (18)
H23D	0.392714	0.271243	0.869063	0.072*	0.473 (18)
C17A	0.4217 (9)	0.1943 (9)	0.9644 (5)	0.0350 (15)	0.527 (18)
H17A	0.310396	0.216499	0.973800	0.042*	0.527 (18)
H17B	0.382241	0.070510	0.950344	0.042*	0.527 (18)
C17B	0.488 (2)	0.1633 (11)	0.9496 (7)	0.060 (3)	0.473 (18)
H17C	0.552732	0.089741	0.934860	0.072*	0.473 (18)
H17D	0.351948	0.090610	0.946041	0.072*	0.473 (18)
C18	0.5744 (3)	0.2870 (3)	1.04731 (17)	0.0464 (6)	
H18A	0.630209	0.212733	1.062407	0.056*	0.527 (18)
H18B	0.524111	0.325978	1.101486	0.056*	0.527 (18)
H18C	0.479995	0.315917	1.077251	0.056*	0.473 (18)
H18D	0.632966	0.241116	1.087953	0.056*	0.473 (18)
C19	0.7190 (3)	0.4355 (3)	1.02201 (16)	0.0432 (6)	
H19A	0.736439	0.542873	1.065185	0.052*	
H19B	0.841615	0.429460	1.025572	0.052*	

### Atomic displacement parameters (Å^2^)

**Table:**

	*U*^11^	*U*^22^	*U*^33^	*U*^12^	*U*^13^	*U*^23^
S1	0.0178 (2)	0.0277 (3)	0.0187 (2)	0.00755 (18)	0.00495 (16)	0.00898 (17)
S2	0.0177 (2)	0.0326 (3)	0.0258 (2)	0.0071 (2)	0.00239 (17)	0.00938 (19)
O1	0.0207 (6)	0.0323 (8)	0.0260 (7)	0.0062 (6)	0.0074 (5)	0.0127 (6)
O2	0.0340 (7)	0.0270 (7)	0.0193 (6)	0.0155 (6)	0.0014 (5)	0.0059 (5)
O3	0.0381 (8)	0.0300 (8)	0.0192 (6)	0.0180 (6)	−0.0001 (5)	0.0061 (6)
O4	0.0537 (11)	0.0545 (11)	0.0353 (9)	−0.0034 (9)	0.0028 (8)	0.0182 (8)
N1	0.0194 (7)	0.0214 (8)	0.0166 (7)	0.0071 (6)	0.0036 (5)	0.0057 (6)
C1	0.0263 (9)	0.0293 (10)	0.0216 (9)	0.0131 (8)	0.0018 (7)	0.0066 (7)
C2	0.0180 (9)	0.0275 (10)	0.0253 (9)	0.0076 (8)	0.0024 (7)	0.0034 (8)
C3	0.0198 (9)	0.0225 (9)	0.0232 (9)	0.0078 (7)	0.0070 (7)	0.0066 (7)
C4	0.0203 (8)	0.0209 (9)	0.0193 (8)	0.0099 (7)	0.0042 (7)	0.0031 (7)
C5	0.0186 (8)	0.0215 (9)	0.0258 (9)	0.0070 (7)	0.0028 (7)	0.0050 (7)
C6	0.0262 (9)	0.0234 (10)	0.0253 (9)	0.0099 (8)	0.0052 (7)	0.0090 (7)
C7	0.0221 (9)	0.0193 (9)	0.0213 (9)	0.0071 (7)	0.0058 (7)	0.0060 (7)
C8	0.0212 (9)	0.0176 (9)	0.0202 (8)	0.0066 (7)	0.0033 (7)	0.0034 (7)
C9	0.0221 (9)	0.0326 (11)	0.0214 (9)	0.0123 (8)	0.0055 (7)	0.0100 (8)
C10	0.0192 (8)	0.0191 (9)	0.0206 (8)	0.0059 (7)	0.0052 (7)	0.0047 (7)
C11	0.0203 (9)	0.0189 (9)	0.0191 (8)	0.0062 (7)	0.0059 (7)	0.0047 (7)
C12	0.0213 (9)	0.0203 (9)	0.0184 (8)	0.0073 (7)	0.0032 (7)	0.0035 (7)
C13	0.0230 (9)	0.0201 (9)	0.0169 (8)	0.0085 (7)	0.0042 (7)	0.0038 (7)
C14	0.0235 (9)	0.0240 (10)	0.0192 (8)	0.0099 (8)	0.0039 (7)	0.0086 (7)
C15	0.0195 (8)	0.0255 (10)	0.0192 (8)	0.0080 (7)	0.0042 (7)	0.0086 (7)
C16	0.0499 (15)	0.0590 (18)	0.0409 (14)	0.0004 (13)	0.0085 (12)	0.0025 (12)
C17A	0.030 (3)	0.028 (3)	0.048 (3)	0.012 (2)	0.012 (2)	0.012 (2)
C17B	0.062 (7)	0.034 (4)	0.054 (4)	−0.006 (3)	−0.012 (4)	0.014 (3)
C18	0.0434 (13)	0.0515 (15)	0.0429 (13)	0.0158 (12)	0.0087 (10)	0.0195 (11)
C19	0.0423 (13)	0.0387 (13)	0.0438 (13)	0.0125 (11)	−0.0052 (10)	0.0158 (10)

### Geometric parameters (Å, º)

**Table:**

S1—C13	1.7485 (18)	C9—H9A	0.9800
S1—C11	1.7580 (18)	C9—H9B	0.9800
S2—C13	1.6391 (18)	C9—H9C	0.9800
O1—C12	1.211 (2)	C10—C11	1.350 (2)
O2—C15	1.218 (2)	C10—H10	0.9500
O3—C15	1.314 (2)	C11—C12	1.481 (2)
O3—H3O	0.92 (3)	C14—C15	1.506 (2)
O4—C16	1.401 (3)	C14—H14A	0.9900
O4—C19	1.416 (3)	C14—H14B	0.9900
N1—C13	1.368 (2)	C16—C17B	1.414 (8)
N1—C12	1.400 (2)	C16—C17A	1.522 (7)
N1—C14	1.455 (2)	C16—H23A	0.9900
C1—C6	1.387 (3)	C16—H23B	0.9900
C1—C2	1.389 (3)	C16—H23C	0.9900
C1—H1	0.9500	C16—H23D	0.9900
C2—C3	1.386 (3)	C17A—C18	1.492 (7)
C2—H2	0.9500	C17A—H17A	0.9900
C3—C4	1.402 (2)	C17A—H17B	0.9900
C3—H3	0.9500	C17B—C18	1.549 (9)
C4—C5	1.405 (3)	C17B—H17C	0.9900
C4—C7	1.465 (2)	C17B—H17D	0.9900
C5—C6	1.388 (3)	C18—C19	1.499 (3)
C5—H5	0.9500	C18—H18A	0.9900
C6—H6	0.9500	C18—H18B	0.9900
C7—C8	1.357 (2)	C18—H18C	0.9900
C7—H7	0.9500	C18—H18D	0.9900
C8—C10	1.450 (2)	C19—H19A	0.9900
C8—C9	1.504 (2)	C19—H19B	0.9900
			
C13—S1—C11	92.63 (8)	C15—C14—H14A	109.5
C15—O3—H3O	111.2 (18)	N1—C14—H14B	109.5
C16—O4—C19	109.06 (19)	C15—C14—H14B	109.5
C13—N1—C12	117.09 (14)	H14A—C14—H14B	108.1
C13—N1—C14	122.23 (14)	O2—C15—O3	124.76 (17)
C12—N1—C14	120.44 (14)	O2—C15—C14	122.99 (16)
C6—C1—C2	119.91 (17)	O3—C15—C14	112.25 (15)
C6—C1—H1	120.0	O4—C16—C17B	109.1 (4)
C2—C1—H1	120.0	O4—C16—C17A	106.1 (3)
C3—C2—C1	119.45 (17)	O4—C16—H23A	110.5
C3—C2—H2	120.3	C17A—C16—H23A	110.5
C1—C2—H2	120.3	O4—C16—H23B	110.5
C2—C3—C4	121.68 (17)	C17A—C16—H23B	110.5
C2—C3—H3	119.2	H23A—C16—H23B	108.7
C4—C3—H3	119.2	O4—C16—H23C	109.9
C3—C4—C5	117.91 (16)	C17B—C16—H23C	109.9
C3—C4—C7	117.98 (16)	O4—C16—H23D	109.9
C5—C4—C7	124.08 (16)	C17B—C16—H23D	109.9
C6—C5—C4	120.33 (17)	H23C—C16—H23D	108.3
C6—C5—H5	119.8	C18—C17A—C16	103.6 (4)
C4—C5—H5	119.8	C18—C17A—H17A	111.0
C1—C6—C5	120.68 (17)	C16—C17A—H17A	111.0
C1—C6—H6	119.7	C18—C17A—H17B	111.0
C5—C6—H6	119.7	C16—C17A—H17B	111.0
C8—C7—C4	130.30 (16)	H17A—C17A—H17B	109.0
C8—C7—H7	114.9	C16—C17B—C18	106.1 (6)
C4—C7—H7	114.9	C16—C17B—H17C	110.5
C7—C8—C10	116.00 (16)	C18—C17B—H17C	110.5
C7—C8—C9	124.99 (16)	C16—C17B—H17D	110.5
C10—C8—C9	118.99 (15)	C18—C17B—H17D	110.5
C8—C9—H9A	109.5	H17C—C17B—H17D	108.7
C8—C9—H9B	109.5	C17A—C18—C19	105.8 (3)
H9A—C9—H9B	109.5	C19—C18—C17B	99.5 (4)
C8—C9—H9C	109.5	C17A—C18—H18A	110.6
H9A—C9—H9C	109.5	C19—C18—H18A	110.6
H9B—C9—H9C	109.5	C17A—C18—H18B	110.6
C11—C10—C8	130.49 (16)	C19—C18—H18B	110.6
C11—C10—H10	114.8	H18A—C18—H18B	108.7
C8—C10—H10	114.8	C19—C18—H18C	111.9
C10—C11—C12	119.76 (16)	C17B—C18—H18C	111.9
C10—C11—S1	130.59 (14)	C19—C18—H18D	111.9
C12—C11—S1	109.55 (12)	C17B—C18—H18D	111.9
O1—C12—N1	122.83 (16)	H18C—C18—H18D	109.6
O1—C12—C11	127.15 (16)	O4—C19—C18	107.68 (19)
N1—C12—C11	110.02 (14)	O4—C19—H19A	110.2
N1—C13—S2	126.39 (13)	C18—C19—H19A	110.2
N1—C13—S1	110.58 (12)	O4—C19—H19B	110.2
S2—C13—S1	123.03 (11)	C18—C19—H19B	110.2
N1—C14—C15	110.82 (14)	H19A—C19—H19B	108.5
N1—C14—H14A	109.5		
			
C6—C1—C2—C3	−0.1 (3)	S1—C11—C12—O1	−179.34 (16)
C1—C2—C3—C4	−1.5 (3)	C10—C11—C12—N1	−175.57 (16)
C2—C3—C4—C5	2.3 (3)	S1—C11—C12—N1	1.19 (18)
C2—C3—C4—C7	−179.45 (16)	C12—N1—C13—S2	176.88 (13)
C3—C4—C5—C6	−1.6 (3)	C14—N1—C13—S2	2.6 (2)
C7—C4—C5—C6	−179.72 (16)	C12—N1—C13—S1	−3.58 (19)
C2—C1—C6—C5	0.8 (3)	C14—N1—C13—S1	−177.86 (13)
C4—C5—C6—C1	0.1 (3)	C11—S1—C13—N1	3.52 (13)
C3—C4—C7—C8	153.36 (19)	C11—S1—C13—S2	−176.92 (12)
C5—C4—C7—C8	−28.5 (3)	C13—N1—C14—C15	83.7 (2)
C4—C7—C8—C10	178.50 (17)	C12—N1—C14—C15	−90.43 (19)
C4—C7—C8—C9	−2.9 (3)	N1—C14—C15—O2	12.6 (2)
C7—C8—C10—C11	−175.15 (18)	N1—C14—C15—O3	−167.93 (14)
C9—C8—C10—C11	6.1 (3)	C19—O4—C16—C17B	−1.1 (9)
C8—C10—C11—C12	178.17 (17)	C19—O4—C16—C17A	27.9 (4)
C8—C10—C11—S1	2.2 (3)	O4—C16—C17A—C18	−26.5 (6)
C13—S1—C11—C10	173.64 (18)	O4—C16—C17B—C18	18.8 (13)
C13—S1—C11—C12	−2.65 (13)	C16—C17A—C18—C19	15.4 (6)
C13—N1—C12—O1	−177.95 (16)	C16—C17B—C18—C19	−27.6 (12)
C14—N1—C12—O1	−3.6 (3)	C16—O4—C19—C18	−17.9 (3)
C13—N1—C12—C11	1.5 (2)	C17A—C18—C19—O4	0.3 (4)
C14—N1—C12—C11	175.94 (14)	C17B—C18—C19—O4	27.2 (7)
C10—C11—C12—O1	3.9 (3)		

### Hydrogen-bond geometry (Å, º)

**Table:**

*D*—H···*A*	*D*—H	H···*A*	*D*···*A*	*D*—H···*A*
O3—H3*O*···O2^i^	0.92 (3)	1.73 (3)	2.6440 (18)	175 (3)
C14—H14*B*···O4	0.99	2.26	3.127 (2)	145
C2—H2···O1^ii^	0.95	2.51	3.389 (2)	154

Symmetry codes: (i) −*x*+2, −*y*+2, −*z*+2; (ii) −*x*, −*y*+1, −*z*+1.
